# Assessment of Biochemical and Densitometric Markers of Calcium-Phosphate Metabolism in the Groups of Patients with Multiple Sclerosis Selected due to the Serum Level of Vitamin D_3_

**DOI:** 10.1155/2018/9329123

**Published:** 2018-08-23

**Authors:** Natalia Niedziela, Krystyna Pierzchała, Jolanta Zalejska-Fiolka, Jacek T. Niedziela, Ewa Romuk, Magdalena Torbus-Paluszczak, Monika Adamczyk-Sowa

**Affiliations:** ^1^Department of Neurology, SMDZ in Zabrze, Medical University of Silesia in Katowice, ul. 3-go Maja 13-15, 41-800 Zabrze, Poland; ^2^Department of Biochemistry, SMDZ in Zabrze, Medical University of Silesia in Katowice, ul. Jordana 19, 41-808 Zabrze, Poland; ^3^3rd Department of Cardiology, SMDZ in Zabrze, Medical University of Silesia, Katowice, Silesian Centre for Heart Disease, Zabrze, Poland

## Abstract

**Background:**

In addition to the widely known effect of vitamin D_3_ (vitD_3_) on the skeleton, its role in the regulation of the immune response was also confirmed.

**Aim:**

The assessment of biochemical and densitometric markers of calcium-phosphate metabolism in the groups of patients with relapsing-remitting multiple sclerosis (RRMS) selected due to the serum level of vitamin D_3_.

**Methods:**

The concentrations of biochemical markers and indices of lumbar spine bone densitometry (DXA) were determined in 82 patients divided into vitamin D_3_ deficiency (VitDd), insufficiency (VitDi), and normal vitamin D_3_ level (VitDn) subgroups.

**Results:**

The highest level of the parathyroid hormone (PTH) and the highest prevalence of hypophosphatemia and osteopenia were demonstrated in VitDd group compared to VitDi and VitDn. However, in VitDd, VitDi, and VitDn subgroups no significant differences were observed in the levels of alkaline phosphatase (ALP) and ionized calcium (Ca^2+^) and in DXA indices. A negative correlation was observed between the level of vitamin D_3_ and the Expanded Disability Status Scale (EDSS) in the whole MS group. The subgroups were significantly different with respect to the EDSS scores and the frequency of complaints related to walking according to the EQ-5D.

**Conclusions:**

It is necessary to assess calcium-phosphate metabolism and supplementation of vitamin D_3_ in RRMS patients. The higher the clinical stage of the disease assessed with the EDSS, the lower the level of vitamin D_3_ in blood serum. Subjectively reported complaints related to difficulties with walking were reflected in the EDSS in VitDd patients.

## 1. Introduction

Progressive disability is one of the major clinical problems in multiple sclerosis (MS) patients [1]. Neurological deficits develop due to inflammatory demyelinating lesions in the central nervous system (CNS) and are the result of damage to the myelin sheath and nerve cell axons [2–4]. This disorder contributes to the increase in functional impairment and the deterioration of the quality of life of young people.

The complex role of immune, genetic and environmental factors is stressed in the formation of demyelinating lesions and in the course of the disease [5, 6]. Reduced exposure to ultraviolet (UVB) radiation and, as a result, vitaminD_3_ (vitD_3_) deficiency plays a key role in the etiopathogenesis of MS [7–9]. Low level of vitD_3_ is an early predictor of the both disease progression [10] and relapses [11]. The contribution of vitD_3_ to the regulation of calcium-phosphate metabolism is well known [12]. Its most important extracalcemic activity is limiting of autoimmune progression in MS, mainly due to increased anti-inflammatory response [12–14].

Vitamin D_3_ with the parathyroid hormone (PTH), phosphorus (P), and ionized calcium (Ca^2+^) are responsible for calcium-phosphate metabolism and normal bone status. Secretion of PTH occurs in response to reduced Ca^2+^ level and its targeted effect is related to increased Ca^2+^ reabsorption in the distal renal tubule with a simultaneous release of Ca^2+^ from bones and osteoclastic bone resorption. In addition, PTH enhances the activity of 1-*α* hydroxylase in the renal proximal tubule responsible for the synthesis of calcitriol, which is an active metabolite of vitD_3_. At the same time, through its anabolic activity, PTH stimulates bone formation greater than resorption. Concomitantly, PTH increases phosphaturia and decreases P in blood serum [15]. Vitamin D_3_ also facilitates bone mineralization and its deficiency contributes to the disturbance in remodeling processes with a simultaneous delay in bone growth [16]. Inadequate dietary intake of calcium contributes to increased bone turnover due to vitD_3_ and PTH [17].

In cases of impaired calcium-phosphate metabolism, the loss of bone tissue is observed, which contributes to the development of osteoporosis (OS). Osteoporosis is much more prevalent in MS patients compared to the general population, constituting an important risk factor for fractures associated with increased morbidity and mortality [18]. Disseminated demyelinating lesions of the CNS result in problems with vision, balance, and coordination, which is inextricably linked to an increased susceptibility to falls and fractures in MS. The potential causes of the increasing risk of OS in MS patients include MS-related weakness and disability, decreased exposure to UVB, chronic inflammatory process, and glucocorticosteroid (GC) therapy during relapses [19].

The aim of the study was to assess biochemical parameters of calcium-phosphate metabolism, densitometric indices, and the style and quality of life in relapsing-remitting multiple sclerosis (RRMS) patients with deficiency, insufficiency, and normal levels of vitD_3_.

## 2. Material and Methods

### 2.1. Study Group

All consecutive patients with RRMS were prospectively recruited from the Department of Neurology in Zabrze, Medical University of Silesia, Katowice, Poland. The study was done from December 2013 to March 2014. All patients enrolled in the study were divided depending on serum levels of vitD_3_:

RRMS VitD deficiency subgroup (VitDd): serum vitD_3_ level <20 ng/ml

RRMS VitD insufficiency subgroup (VitDi): serum vitD_3_ level of 20-30 ng/ml

RRMS VitD normal subgroup (VitDn): serum vitD_3_ level > 30 ng/ml

### 2.2. Inclusion and Exclusion Criteria

The inclusion criteria were as follows: age ≥ 18 years, RRMS diagnosed according to the McDonald criteria (2010), RRMS patients treated with disease-modifying therapy (DMT) or patients without immunomodulatory treatment prior to the study, Expanded Disability Status Scale (EDSS) ≤ 5, Caucasian race, residence on the territory of the Upper Silesia, and written informed consent for participation in the study. The exclusion criteria were as follows: clinical disease other than RRMS, MS relapse and GC therapy within one month before the enrollment, OS diagnosed prior to the study, the intake of medications affecting calcium-phosphate metabolism for < 6 months before the enrollment, intestinal malabsorption syndromes, conditions that may be associated with hypercalcemia (tuberculosis, sarcoidosis), heart failure, arrhythmias, liver, kidney, and thyroid function disorders, fracture of lumbar vertebrae in history, hematological disorders diagnosed during the last 3 months prior to the study, pregnancy or breast feeding, indoor stay only or underground work, and travel to another climate zone <6 months before the study.

### 2.3. Study Protocol

The diagnostic procedures were conducted during the morning medical visit and included the following: physical examination, medical history extended by an epidemiological survey, the panel of biochemistry blood tests, and lumbar spine bone densitometry (DXA). The clinical stage of MS was assessed using the EDSS performed by an experienced EDSS rater.

### 2.4. Densitometric Analysis

The densitometric analysis was done using DXA (the Explorer S/N 91755). Lumbar vertebral bone mass was assessed (anterior-posterior L2-L4). This method allowed the measurement of bone mineral density (BMD) and T-score and Z-score indices. Osteoporosis was diagnosed based on the current WHO diagnostic criteria (1994), i.e., for T-score <-2.5, osteopenia (OP) for T-score between -2.5 and -1.0. Normal bone status was determined for T-score> -1.0.

### 2.5. Laboratory Assays

Fasting serum samples, collected between 7am and 8am, were used for biochemical tests. Thirty minutes after collection, the samples were centrifuged, frozen, and stored at -80°C until the markers of calcium-phosphate metabolism were determined.

#### 2.5.1. 25-Hydroxycholecalciferol

25(OH)-Vitamin D direct day enzyme-linked immunosorbent assay (ELISA) Kit (Immundiagnostik AG) was used to determine the level of 25-hydroxycholecalciferol [25(OH)D]. The range of reference values was as follows: normal level (>30 ng/ml; >75 nmol/l), insufficiency (20-30 ng/ml; 50-75 nmol/l), and vitD_3_ deficiency (<20 ng/ml; <50 nmol/l). For the purposes of this study, the abbreviation 25(OH)D was synonymous with vitD_3_ and was used interchangeably.

#### 2.5.2. PTH

The level of PTH was assessed using a third-generation test 1-84 PTH, alternatively known as PTH biointact or total PTH. For this purpose, Elecsys PTH (1-84) Modular Analytics E170 cobas e Roche was used, using the electrochemiluminescence (ECLIA). The following levels were considered normal serum PTH: 14.9 pg/mL-56.9 pg/mL (1.58 pmol/L-6.03 pmol/L). The levels of the examined substance in the samples were calculated automatically by the analyzer (pg/mL = pmol/L x 9.43, and pmol/L = pg/mL x 0.106).

#### 2.5.3. Ca^*2*+^

The measurement of Ca^2+^ in blood serum was done using the SIEMENS RAPIDLAB 1265 with direct ISE. The range of reference values for Ca^2+^ was 1.1-1.35 mmol/l.

#### 2.5.4. P

Phosphate (inorganic) ver. 2 test (Roche/Hitachi cobas c system) was used for quantitative determination of P in serum. The range of normal values was determined between 0.81 and 1.45 mmol/L (2.5-4.5 mg/dL).

#### 2.5.5. Alkaline Phosphatase

Alkaline Phosphatase acc. to IFCC Gen.2 test was used for the quantitative determination of the activity of alkaline phosphatase (ALP) in blood serum. The test is intended for use in Roche/Hitachi cobas c analyzers. The accepted normal values of ALP ranged from 40 to 130 U/L for men and 35 to 105 U/L for women. The analytical activity of ALP and the level of P for each sample were calculated automatically by the Roche/Hitachi cobas c analyzer.

### 2.6. Epidemiological Survey

The survey questionnaire was prepared according to the concept and experience of the authors. It consisted of 62 obligatory items, while 6 items were the components of the quality of life scale according to the Euro Quality of Life-5 Dimension (EQ-5D). The first part of the questions was related to basic personal data and social and economic information. The second part was related to the onset of the underlying disease. Further questions were related to the current MS status (number of relapses hospitalizations, rehabilitation, current and previous use of DMT, and oral and i.v. GCs). The last part of the questionnaire included questions connected with past fractures and the lifestyle.

### 2.7. Statistical Analysis

Descriptive statistics parameters for continuous variables were presented as the arithmetic mean and the standard deviation. Qualitative variables were presented as percentage values. Homogeneity of the continuous variable between subgroups was analyzed using a parametric ANOVA. The Student* t*-test was used to compare 2 subgroups. Group homogeneity with respect to the qualitative variable was analyzed by the chi-squared test using the Yates correction when the expected frequency table included the values < 5. The post hoc analysis was performed using the Tukey test with the Bonferroni correction if significant differences were found in the markers of calcium-phosphate metabolism and the EDSS. The frequencies between the subgroups were compared using the contingency tables and the chi-square test. In the statistical analysis the significance level of p (*α*) <0.05 was adopted. Statistica 10 PL software (StatSoft) was used for statistical calculations.

## 3. Results

Eighty-two patients with RRMS were prospectively enrolled in the study. The mean age of patients was 42.7 ± 10.5 [years]; 73.2% of the patients were women. The mean age at diagnosis of MS was 38.2 ± 10.3 [years], and the number [n] of relapses was 4.37 ± 2.44. The neurological status of all patients assessed by the EDSS [score] was determined at 2.54 ± 0.99. The first symptoms of the disease occurred in 37% of patients in the spring. The number of cycles of* iv. *GCs was 2.19 ± 1.9, while the number of hospitalizations due to the primary disease was 3.15 ± 1.91. Patients on DMT at the time of the study constituted 75.6% of the study group. The most common form of therapy was interferon-*β*1b (INF *β*-1b) (31.7%), and another treatment included interferon-*β*1a (INF *β*-1a) (23.2%), glatiramer acetate (7.3%), and fingolimod or natalizumab (13.4%). The family history of MS was confirmed in 6.1% of patients, while 60% of cases were related to first-line relatives and 40% to second-line relatives. Disability pension due to MS was given to 37% of patients.

**Figure 1:**
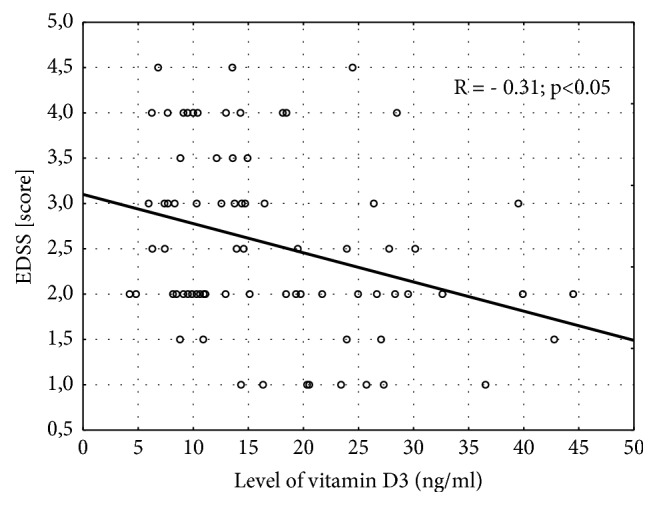
Pearson's linear correlation coefficient between 25-hydroxycholecalciferol (vitD3) and the Expanded Disability Status Scale (EDSS) in multiple sclerosis patients.

There were 56 patients (68%) with VitDd, 19 patients (23%) with VitDi, and 7 patients (9%) with VitDn. The basic characteristics of VitDd, VitDi, and VitDn patients are shown in Table 1. The assessment of the clinical status and treatment was presented in Table 2. In the subgroup of VitDn all patients were treated with DMT; however, no significant differences were observed in the selected subgroups. Significant differences were observed in the total scores measured by the EDSS, and the post hoc analysis confirmed higher EDSS in VitDd versus VitDi (p<0.05). A negative correlation between the level of vitD_3_ and the EDSS was observed in the whole MS group (Figure 1).

The analysis of calcium-phosphate metabolism in the patient population divided depending on the level of vitD_3_ (Table 3) showed that these subgroups were not different with respect to ALP levels (including different sex-related norms) or Ca^2+^ serum levels. However, hypocalcemia was reported in the whole MS group. Significant differences in the prevalence of hypophosphatemia were observed between VitDd, VitDi, and VitDn. In the study group, the mean vitD_3_ level [ng/ml] was 16.9 ± 9.4; elevated PTH levels were observed in 13.0% of patients. No significant differences were observed in the prevalence of hyperPTH between VitDd, VitDi, and VitDn subgroups. Simultaneously, differences in the mean PTH level were confirmed. The post hoc analysis showed that VitDn patients had significantly lower PTH levels compared to VitDd patients (p = 0.04). The prevalence of OS in all MS patients was estimated at 11.4%, OP at 40.5%, while in the lumbar spine the mean T-score value was 0.9 ± 1.3, Z-score was -0.5 ± 1.2, and BMD [g/cm^2^] was 0.9 ± 0.1. No significant difference was observed in the following: T-score, Z-score, BMD [g/cm^2^], and the prevalence of OS [%] or fractures [%] between VitDd, VitDi, and VitDn. These subgroups, however, were different with respect to the prevalence of OP (Table 4).

**Figure 2:**
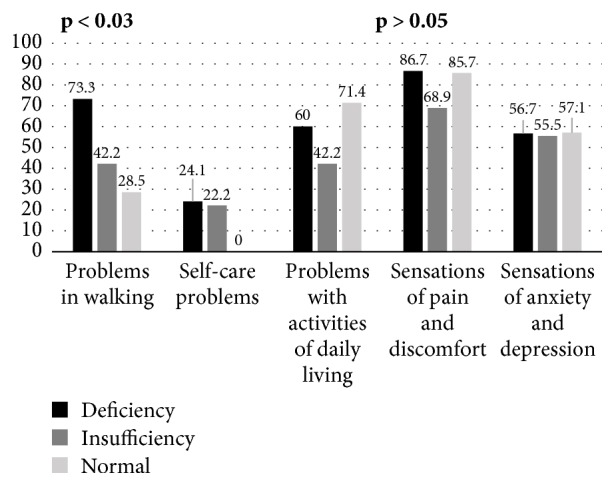
Comparison of the EQ-5D quality of life in the subgroups of patients with deficiency, insufficiency, and normal level of 25-hydroxycholecalciferol.

No differences were observed in the subjective assessment of health status measured by the Euro Quality-Visual Analogue Scale (EQ-VAS) in VitDd, VitDi, and VitDn patients (64.6 ± 16.6 versus 67.1 ± 19.2 versus 72.9 ± 18.7, respectively; p = 0.40). Significant differences were noted with respect to problems in walking in the above subgroups. However, there were no differences in the other components of the EQ-5D scale (Figure 2).

The analyzed subgroups were not different in terms of particular aspects of lifestyle, i.e., smoking frequency before and at the time of the study (%) (50.0 versus 38.9 versus 28.6; p = 0.47), alcohol consumption before and at the time of the study (81.8 versus 78.9 versus 100.0; p = 0.44), physical activity at least 30 minutes during the day (19.0 versus 42.1 versus 42.9; p = 0.10), the use of sunbeds (35.7 versus 42.1 versus 57.1; p = 0.53), births between October and March (41.1 versus 63.1 versus 42.9; p = 0.25), or dietary habits (p> 0.05) for VitDd, VitDi, and VitDn patients, respectively.

## 4. Discussion

The analysis of calcium-phosphate metabolism clearly indicates that PTH is the only marker, whose profile is unquestionably different in VitDd, VitDi, and VitDn subgroups. No differences were observed in the mean levels of other biochemical parameters. Significant differences in the prevalence of hypophosphatemia were due to the fact that patients from VitDi and VitDn did not present with hypophosphatemia. Under physiological conditions, vitD_3_ enhances the absorption of P in the gastrointestinal tract. Therefore, it can be assumed that hypophosphatemia appears only in the case of deficiency of vitD_3_. Additional inclusion of phosphaturic activity of the fibroblast growth factor (FGF-23) and the determination of its coreceptor (Klotho protein) in RRMS patients would have had an impact on the conclusions [20]. Both serum P and Ca2+ levels are largely dependent on their dietary intake and in the questionnaire assessment patients probably responded in accordance with dietary requirements, not necessarily in accordance with real eating habits. Despite the fact that Ca^2+^ level is believed to best reflect the calcium metabolism status in the body, as much as 40% of calcium which circulates in the blood is albumin-bound [21]. In turn, the determination of levels of albumins and total protein which has an impact on the active fraction of total calcium could have modified the results. The lack of differences in the levels of ALP between VitDd, VitDi, and VitDn is consistent with the study conducted in patients who did not use DMT [22].

The detailed assessment confirmed that VitDd patients had significantly higher serum PTH levels compared to VitDn patients (p = 0.04). A similar significant correlation between the level of vitD_3_ and PTH was confirmed by Tulay et al [23]. To some extent, the above relationship results from the physiology of the endocrine system, which under optimal conditions provides normal calcium-phosphate metabolism. Ca^2+^ ions participate in maintaining this balance, and their low level in serum is the main factor which stimulates PTH secretion [16]. The inverse correlation between PTH and vitD_3_ is reported and hence the increased secretion of PTH is observed with the deficiency of vitD_3_ and Ca^2+^. On the other hand, vitD_3_ deficiency was reported in 68% of RRMS patients, while hyperPTH was reported only in 13% of patients. This finding suggests that hypersecretion of PTH appears with a delay compared to the decrease in the level of vitD_3_ in MS patients. At the same time, hyperPTH was not observed in any patient from the VitDn subgroup. Bearing in mind that all patients from VitDn used DMT, it can be assumed that this treatment affected the serum PTH level. However, the final impact of this treatment on the above hormone requires careful and detailed research.

In the entire population of patients with RRMS, OS was confirmed only in 11.4% of patients and OP in 40.5%. These results are consistent with the broad analysis of Gibson et al. Based on the literature review, those researchers documented the occurrence of OP in 26-73% and OS in 5-29% [24]. No significant differences were confirmed in the assessment of the percentage of the prevalence of OS in VitDd, VitDi, and VitDn subgroups, which would probably have occurred in a comparative analysis with the control group. A much higher prevalence of OS is observed in MS patients with respect to the general population [19, 24]. At the same time, the highest percentage (50.9%) of OP was reported in VitDd, which suggests the need to consider vitD_3_ supplementation. Not only does its deficiency result in the disorders of calcium-phosphate metabolism but it is also a risk factor of OP and, consequently, OS. Noteworthy, the diagnosis of OS in this study was established based on the T-score <-2.5. However, according to the WHO recommendations (1994), consideration of T-score in the diagnosis of the disease is only acceptable for postmenopausal women and for men over the age of 50. In patients <50 years of age, OS should be diagnosed on the basis of Z-score ≤ -2, with the cooccurrence of osteoporotic fracture or the risk factor for secondary OS in history [25, 26]. Based on the assumptions that MS mainly affects young people and as an autoimmune disease it is a risk factor for secondary OS, the diagnosis of the disease with the Z-score seems to be more precise. However, the T-score was considered in the diagnosis of OS in many studies on bone status assessment in MS patients [23, 27–29].

In VitDd, VitDi, and VitDn subgroups no significant differences were found in T-score, Z-score, or BMD values measured in the lumbar spine (p> 0.05). Due to the superior function of vitD3 in the maintenance of the balance of calcium-phosphate metabolism in MS patients, a potential risk of decreased BMD value is observed [30, 31]. However, many studies did not prove the relationship between deficiency of vitamin D_3_ and reduced BMD [24, 32–35], as opposed to other reports [23, 36]. In the past, lower BMD values were observed in MS patients as compared to the control group [23, 27, 37]. A negative correlation between BMD and the EDSS was also reported [23, 28, 32], which was probably secondary to the impaired ability determining low BMD values. It was confirmed that one of the determinants of BMD is disease duration [28] and* iv*. GCs [32]. VitDd, VitDi, and VitDn subgroups were not different with respect to the number of cycles of* iv*. GC therapy associated with MS (p> 0.05). Zorzon et al. excluded the increasing risk of OS in MS patients which was the effect of repeated GC cycles [38]. The relationship between BMD and iv. GCs in MS patients was confirmed by Tyblova et al. [39], as opposed to other studies [27, 28].

In terms of the etiopathogenesis of MS, we analyzed the factors related to the lifestyle, i.e., smoking, obesity, eating habits, and alcohol consumption. Tobacco smoking is an independent risk factor for the development of MS [40] while recent studies have demonstrated that the consumption of alcohol over 15 g/day is associated with a lower risk of developing the disease [41]. In turn, the so-called Western lifestyle, based on a diet containing high animal fat or salt, and BMI above 27 kg/m^2^ are another risk factor for the development of MS [42, 43]. The problem of tobacco smoking, diet, and alcohol consumption were included in the present study in the context of vitD_3_ level, which makes this issue even more complex and difficult to interpret unambiguously. We did not also confirm the correlation between the autumn and winter months of birth and the presence of insufficiency, deficiency, or normal levels of vitD_3_. However, some MS studies report an increased risk for individuals born in the spring and summer months compared to the autumn and winter months [44, 45].

The neurological status of the study group was assessed based on the EDSS, considering the assessment of ambulation determined in long-term clinical trials in MS patients [46–48]. Despite the EDSS score ≤ 5 established in the inclusion criteria, we observed differences in the total EDSS score between VitDd, VitDi, and VitDn. In our study, lower serum levels of vitD_3_ were associated with a higher degree of disability measured by the EDSS. A similar negative correlation between the above parameters was reported by van der Mei et al. who postulated that EDSS score > 3 in MS patients was associated with greater vitD_3_ deficiency [49]. The similar relationship was also reported in 2012 [50], while Smolders et al. suggested that vitD_3_ not only was associated with the degree of disability due to MS but could also be a predictor of a higher score in EDSS [51]. On the other hand, Yildiz et al. did not confirm significant differences in the levels of vitD_3_ and the EDSS between patients with low and high disease activity [52]. Studies from 2013 and 2015 did not confirm the relationship between the level of vitD_3_ and the EDSS [53, 54]. Mowry et al. found the above relationship unclear [55]. Too low a level of vitD_3_ results in abnormal bone mineralization and, consequently, problems in walking. Considering the fact that patients with higher EDSS scores are less likely to stay outdoors (hence decreased dermal synthesis of vitD_3_), the open question remains whether a direct correlation between low levels of vitD_3_ and development of disability can be taken into account.

In VitDd, VitDi, and VitDn, significant differences were reported only in one component of the EQ-5D, i.e., reported problems in walking which were most common in VitDd. After the comparison of a subjective assessment of gait disturbances with the results of the EDSS, it can be concluded that patients with VitDd significantly more frequently reported problems in walking with a simultaneous occurrence of significant differences in the EDSS between VitDd, VitDi, and VitDn. Considering that only MS patients with the EDSS scores ≤ 5 were included in the study, it can be assumed that the low serum level of vitD_3_ in MS patients is most likely the cause of the gradually progressive weakness experienced by patients even with slight ambulation deficits assessed in the EDSS. The mechanism of the above phenomenon can be explained by deficient bone mineralization due to vitD_3_ deficiency and, consequently, a gradual increase in problems in walking. In addition, a low level of vitD_3_ correlates with the disorders of many immune processes which are initially impaired by an autoimmune background of MS. The analysis of the EQ-5D results clearly indicates that the quality of life is impaired in MS patients which is consistent with the earlier results obtained in the MS population in Poland [56].

There are still no established guidelines to perform DXA in MS patients which define whether and when the implementation of appropriate treatment of vitD_3_ or Ca^2+^ is absolutely necessary. While Hearn et al. suggested DXA screening only for patients with a high degree of disability [19], Kampman et al. indicated the necessity for the examination at 3-5-year intervals [57]. Considering a high risk of fractures in MS patients, it appears that predicting 10-year fracture risk using the Fracture Risk Assessment (FRAX) requires a reliable modification and the creation of an optimal diagnostic tool. The success of the above assumptions is also due to thorough patient education including drug therapy and lifestyle assessment (daily exposure to UVB, physical activity, diet, etc.).

## 5. Study Limitations

Individual daily exposure to UVB was not assessed, which could probably have determined the levels of vitD_3_ more precisely. After the storage of the blood samples at -80°C the quantitative determination of the activity of alkaline phosphatase (ALP) might have been inadequate. DXA applied to different body regions (distal epiphysis of the forearm, femoral neck) would have allowed bone status assessment in RRMS patients. Due to a small number of patients enrolled in the study, the obtained results should only be regarded as preliminary.

## 6. Conclusions

We confirmed a high percentage of abnormal levels of vitD_3_ in RRMS patients. Lower levels of vitD_3_ were associated with higher serum PTH levels and a higher prevalence of OP without significant differences in the markers of densitometric assessment of the bone status in patients with RRMS.

The higher the clinical stage of the disease assessed with the EDSS, the lower the level of vitamin D_3_ in blood serum. The problems in walking measured by the EQ-5D scale in vitD_3_-deficient patients were reflected in the objective assessment with the EDSS.

In RRMS patient group, calcium and phosphate metabolism should be monitored and 25-hydroxycholecalciferol should be supplemented, considering the early stages of the disease.

## Figures and Tables

**Table 1:** Basic characteristics in the subgroups of patients with deficiency, insufficiency, and normal level of 25-hydroxycholecalciferol.

Parameter	VitDd n=56 (68%)	VitDi n=19 (23%)	VitDn n=7 (9%)	p
Age [years]	43.0 ± 10.1	41.9 ±11.8	43.1 ±11.3	0.94
Sex [% female]	69.6	78.9	85.7	0.54
Height [m]	1.68 ± 0.1	1.68 ± 0.1	1.68 ± 0.08	0.93
Body weight [kg]	68.5 ± 18.3	72.5 ± 15.9	71.6 ± 19.7	0.49
BMI [kg/m^2^]	24.3 ± 6.4	25.5 ± 4.5	25.2 ± 5.4	0.31

VitD_3_-25-hydroxycholecalciferol, VitDd: vitamin D_3_ deficiency, VitDi: vitamin D_3_ insufficiency, VitDn: normal vitamin D_3_, and BMI: body mass index.

**Table 2:** The assessment of the clinical status and immunomodulatory treatment in the subgroups of patients with deficiency, insufficiency, and normal level of 25-hydroxycholecalciferol.

Parameter	VitDd n=56 (68%)	VitDi n=19 (23%)	VitDn n=7 (9%)	p
EDSS [score]	2.8 ± 0.9	2.0 ± 2.35	2.0 ± 2.3	**0.005**
Total number of relapses [n]	4.6 ± 2.5	4.0 ± 2.3	3.6 ± 2.3	0.42
Number of relapses per year [n]	2.66 ± 13.4	5.97 ± 23.0	0.6 ± 0.6	0.52
Hospitalizations due to MS [n]	3.44 ± 2.06	2.63 ±1.42	2.29 ±1.38	0.16
*iv. *GCs [n]	2.4 ± 2.1	1.8 ± 1.5	1.6 ± 1.1	0.56
Rehabilitation due to MS [%]	60.7	42.1	42.9	0.30
Treatment [%]:	-	-	-	-
(i) INF-*β*1a [%]	21.4	26.3	28.6	0.86
(ii) INF-*β*1b [%]	26.8	36.8	57.1	0.23
(iii) Copaxone [%]	8.9	5.3	0	0.65
(iv) Gilenya/Tysabri	14.2	10.5	14.3	0.92
INF- *β* if ever [%]	69.6	73.7	100.0	0.24

VitD_3_-25-hydroxycholecalciferol, VitDd: vitamin D_3_ deficiency, VitDi: vitamin D_3_ insufficiency, VitDn: normal vitamin D_3,_ EDSS: Expanded Disability Status Scale; INF-*β*: interferon beta, MS: multiple sclerosis, *iv*. GCs: intravenous glucocorticoids.

**Table 3:** Comparison of biochemical markers of calcium-phosphate metabolism in the subgroups of patients with deficiency, insufficiency, and normal level of 25-hydroxycholecalciferol.

Parameter	VitDd n=30	VitDi n=45	VitDn n=7	p
ALP [U/L]	71.8 ± 35.3	61.7 ± 18.0	64.6 ± 14.8	0.22
abnormal ALP [%]	7.1	5.3	0	0.75
P [mmol/l]	0.95 ± 0.15	0.96 ± 0.13	0.98 ± 0.14	0.90
Hypophosphatemia [%]	21.4	0	0	**0.039**
Ca^2+^ [mmol/l]	0.92 ± 0.09	0.94 ± 0.04	0.91 ± 0.04	0.42
hypocalcemia [%]	100.0	100.0	100.0	1.0
PTH [pg/ml]	40.7 ± 14.1	34.4 ±15.4	27.3 ± 7.7	**0.019**
hyperPTH [%]	16.1	10.5	0	0.46

VitD_3_: 25-hydroxycholecalciferol, VitDd: vitamin D_3_ deficiency, VitDi: vitamin D_3_ insufficiency, VitDn: normal vitamin D_3,_ ALP: alkaline phosphatase, P: phosphorus, Ca^2+^: ionized calcium, PTH: parathyroid hormone, HyperPTH: hyperparathormonemia

**Table 4:** Comparison of densitometric markers of calcium-phosphate metabolism in the subgroups of patients with deficiency, insufficiency, and normal level of 25-hydroxycholecalciferol.

Parameter	VitDd n=56 (68%)	VitDi n=19 (23%)	VitDn n=7 (9%)	p
T-Score	-1.11 ± 1.24	-0.53 ± 1.43	-0.71 ± 1.05	0.23
Z-score	-0.74 ±1.23	-0.06 ± 1.20	-0.2 ± 1.42	0.14
BMD [g/cm^2^]	0.94 ± 0.13	1.00 ± 0.16	0.98 ± 0.12	0.25
OP [%]	50.9	21.0	14.3	**0.03**
OS [%]	9.4	15.8	14.3	0.74
Fractures [%]	22.0	41.2	28.6	0.31

VitD3: 25-hydroxycholecalciferol, VitDd: vitamin D_3_ deficiency, VitDi: vitamin D_3_ insufficiency, VitDn: normal vitamin D_3_, BMD: bone mineral density, OP: osteopenia, and OS: osteoporosis

## Data Availability

The data used to support the findings of this study have been deposited in the [NIEDZIELA NATALIA] repository (ORCID ID https://orcid.org/0000-0003-0834-5727).
